# Doxorubicin Encapsulation in Carbon Nanotubes Having Haeckelite or Stone–Wales Defects as Drug Carriers: A Molecular Dynamics Approach

**DOI:** 10.3390/molecules26061586

**Published:** 2021-03-13

**Authors:** Leonor Contreras, Ignacio Villarroel, Camila Torres, Roberto Rozas

**Affiliations:** 1 Laboratorio de Química Computacional y Propiedad Intelectual, Departamento de Ciencias del Ambiente, Facultad de Química y Biología, Universidad de Santiago de Chile, USACH, Avenida Libertador Bernardo O’Higgins 3363, Casilla 40, Correo 33, Santiago 9170022, Chile; roberto.rozas@usach.cl; 2 Departamento de Computación e Informática, Facultad de Ingeniería, Universidad de Santiago de Chile, USACH, Avenida Ecuador 3659, Santiago 9170022, Chile; gatrevolution@gmail.com (I.V.); ctorres@kpitec.com (C.T.)

**Keywords:** carbon nanotubes, Stone–Wales defects, haeckelite defects, doxorubicin encapsulation, drug delivery system, binding free energies, noncovalent interactions, molecular dynamics

## Abstract

Doxorubicin (DOX), a recognized anticancer drug, forms stable associations with carbon nanotubes (CNTs). CNTs when properly functionalized have the ability to anchor directly in cancerous tumors where the release of the drug occurs thanks to the tumor slightly acidic pH. Herein, we study the armchair and zigzag CNTs with Stone–Wales (SW) defects to rank their ability to encapsulate DOX by determining the DOX-CNT binding free energies using the MM/PBSA and MM/GBSA methods implemented in AMBER16. We investigate also the chiral CNTs with haeckelite defects. Each haeckelite defect consists of a pair of square and octagonal rings. The armchair and zigzag CNT with SW defects and chiral nanotubes with haeckelite defects predict DOX-CNT interactions that depend on the length of the nanotube, the number of present defects and nitrogen doping. Chiral nanotubes having two haeckelite defects reveal a clear dependence on the nitrogen content with DOX-CNT interaction forces decreasing in the order 0N > 4N > 8N. These results contribute to a further understanding of drug-nanotube interactions and to the design of new drug delivery systems based on CNTs.

## 1. Introduction

Doxorubicin (DOX), an antineoplasic drug, approved for medical use by the FDA [1,2], has been used for more than 40 years to combat various types of cancers despite the cardiological risks associated with its use. Researchers at the Mayo Clinic [3] have reviewed its mechanism of action and, mainly thanks to the work of Denard et al. and Zhang et al. [4,5], have proposed two alternatives that explain its function. In one of them, DOX would stabilize a complex formed by double-stranded DNA and topoisomerase, which later it would cut both strands of DNA. The alternative is the production of a larger quantity of ceramides which would produce the translocation of a CREB3L-1 protein from the endoplasmic reticulum to the Golgii apparatus. There, some proteases would break the CREB3L-1 protein in such a way that its N-terminal fragment would be translocated to the nucleus where it would direct the DNA transduction, to finally express p21 proteins, which would be those that inhibit tumor growth. Other mechanisms of action of DOX reviewed by Ferreira et al. consider the intercalation of DOX in nuclear DNA and mitochondrial DNA, inhibition of topoisomerase-IIß, and epigenetic factors that involve methylation and deacetylation reactions [6].

In order to increase drug bioavailability and avoid its adverse effects, several types of drug carriers have been used, among which carbon nanotubes (CNTs) have shown to form stable associations with DOX [7]. However, CNTs also exhibit some toxicity problems [8,9,10,11]. Fortunately, CNTs can be functionalized with fragments to increase their water solubility, which prevents them from being deposited as agglomerates in the body. Under certain conditions of concentration and purity CNTs are non-toxic [12]. Additionally, the functionalization of the CNTs facilitates the anchoring of the nanotubes right in the tumor to be attacked. The physicochemical and conductive properties of CNTs give them a versatility of applications in various fields such as electronics, photonics, catalysts, drug carriers, biotechnology, bone tissue engineering and others [13,14,15].

In the current work we are interested in the ability of CNTs to adsorb drugs and transport them to the target site. It is important to determine the structural parameters that facilitate the DOX-CNT association and to allow the development of strong DOX-CNT intermolecular interaction forces, which help to inhibit the drug from being released before reaching its target. Once there, the acidic pH of the tumor environment causes the release of the drug. Indeed, several studies show experimentally that DOX release is favored at pH of 5 or less [16,17,18,19], which is also demonstrated at a theoretical level [20].

Another technologically important characteristic of CNTs is their chirality which significantly modify their conductive properties. For example, armchair (n, n) nanotubes are conductive while zigzag (n, 0) and chiral (n, m) nanotubes are semiconductors except when the value of the difference (n − m) is a multiple of 3, since nanotubes become conductive [21,22,23,24]. The diameter of the nanotube has also been shown to be an important structural point since, depending on the diameter, the degree of curvature of the nanotube can be controlled, which seems to be a decisive factor in the stability of the DOX-CNT associations.

As can be deduced, the chirality and the diameter of the nanotubes are properties that determine their behavior and also the presence of structural defects which can change the chirality [25]. The carbon rings of the nanotube that differ from the hexagons are called defects, and depending on their ordering and distribution in the nanotube they confer different properties. For example, the five- and seven-membered rings, when distributed around the perimeter of the nanotube, constitute defects called bumpy. If they are distributed axially in the nanotube they are called zipper defects [26]. Other four-membered rings, along with eight-membered rings, are called haeckelite defects [22,27]. These defects are formed by the addition of two carbon atoms or ad-dimers. However, there are other defects that are formed by rearrangement of their bonds, such as the Stone–Wales defects formed by a pair of rings of five and eight members [28].

The presence of some of these defects has significant technological importance. For example, zigzag nanotubes in the presence of ad-dimers can induce plastic transformations in a material that would otherwise be brittle [29]. Chiral nanotubes stand out, which in the presence of bumpy defects considerably increase their conductivity over armchair and zigzag nanotubes according to DFT studies considering dispersion-corrected B3LYP-D3 functional [30]. Zigzag nanotubes that contain bumpy defects show greater conductive ability and capacity to be reduced [30]; nitrogen doping increases the conductive ability of armchair nanotubes [30]. In addition, armchair nanotubes with bumpy defects notably increase their ability to adsorb hydrogen with very convenient hydrogen adsorption energy values, for their use in the management of clean energy [30].

CNTs form stable associations with doxorubicin [7]. Various theoretical and molecular dynamics studies predict a high capacity of CNTs as carriers [20,31,32,33,34,35,36] and several molecular dynamics studies of functionalized DOX-CNT systems with various organic groups have contributed to the study of DOX loading and release [37,38,39,40,41,42,43]. Although there are several works on the adsorption of DOX in CNT, there are no studies that report on the optimal structure that a nanotube should have to behave as a DOX nanocarrier. The situation is complicated because there are also no experimental data available on the formation and characterization of DOX-CNT complexes and the determination of their DOX-CNT binding energies. For non-functionalized nanotubes, Wang and Xu [20], systematically studied the adsorption and encapsulation of DOX in armchair nanotubes of different diameters, using the theoretical methods PM6-DH2 and M06-2X in the ONIOM scheme and found that the diameter of the nanotube at which the best DOX encapsulation occurred was 14 Å and corresponded to (10,10) armchair nanotubes. This same behavior was confirmed through a study of molecular dynamics for armchair, zigzag and chiral nanotubes, finding that the strongest DOX-CNT interactions were produced for 14 Å in diameter nanotubes, regardless of chirality [32]. A different situation occurs in the presence of defects in the nanotube. In the case of bumpy defects, a dependence on chirality is observed, since armchair nanotubes with bumpy defects present weaker DOX-CNT interactions than armchair nanotubes without defects. In contrast, bumpy defects in chiral nanotubes favor the DOX-CNT interaction [32].

Several methods of synthesis of CNTs have been reviewed [44]. However, there is a lack of comparative systematic experimental antecedents on this issue, which makes it possible to pose as a valid hypothesis for a theoretical study that the presence of defects in the nanotube, the type and number of defects and their position, also modify the DOX-CNT association properties, along with the chirality and size of the nanotube.

The previous antecedents also lead us to investigate if there is a general trend for some type of nanotube, for example, with chirality or type of defect that accounts for the degree of DOX-CNT association. Our research questions include: (i) how does structural or nitrogen doping defects affect the ability of CNTs as drug carriers, in this case, DOX. (ii) Is the effect produced by the defects the same, regardless of the chirality and the size of the nanotube? (iii) Is there any type of defect that has better characteristics than the others? (iv) How does it affect the number of defects present?

In this work DOX-CNT binding energies are determined for chiral nanotubes with haeckelite defects (with rings of 4 and 8 carbon atoms) and for armchair and zigzag nanotubes with Stone–Wales defects (SW), by means of the MM/PBSA and MM/GBSA methods implemented in the AMBER program of molecular dynamics.

## 2. Results

**Figure 1:**
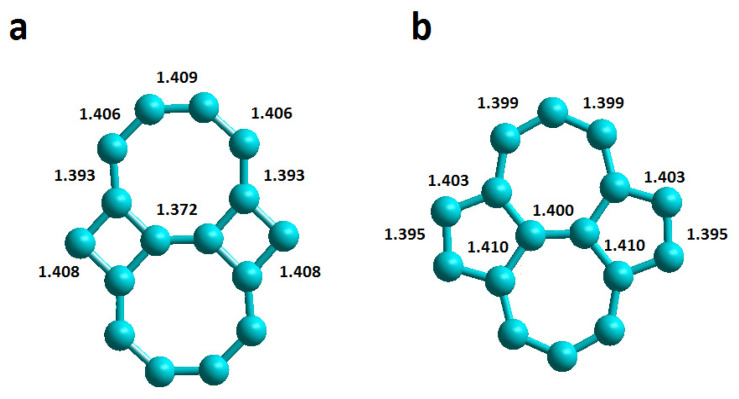
Representation of carbon nanotube structural defects and their C–C bond distances in Å. (**a**) haeckelite defect; (**b**) Stone–Wales defect.

Below are the results obtained by molecular dynamics (MD) simulation for DOX encapsulation systems in chiral CNTs with haeckelite (Hk) defects and also in armchair and zigzag CNTs with Stone–Wales (SW) defects. The Hk defects consist of a pair of rings of 4 and 8 members each, while the SW defects are made up of a pair of rings of 5 and 7 members each, as shown in Figure 1.

### 2.1. Chiral Nanotubes with Hk Defects

**Figure 2:**
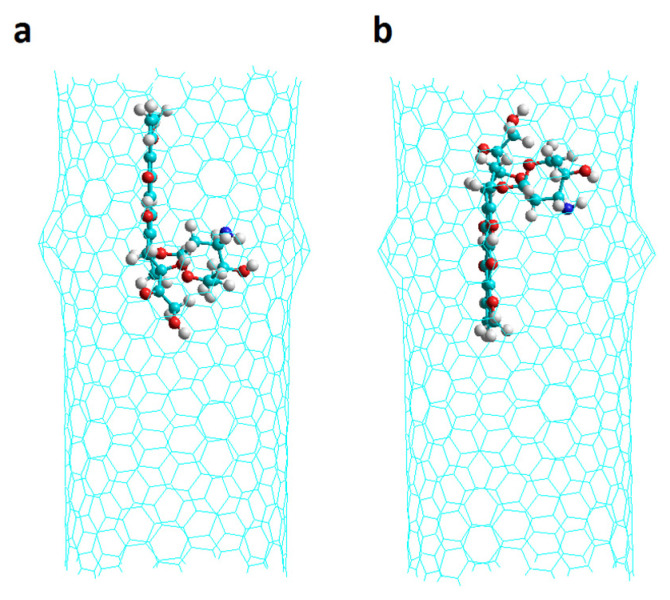
DOX orientation when encapsulated in a chiral nanotube having two haeckelite defects. (**a**) v1 orientation; (**b**) v2 orientation. Lateral views.

**Figure 3:**
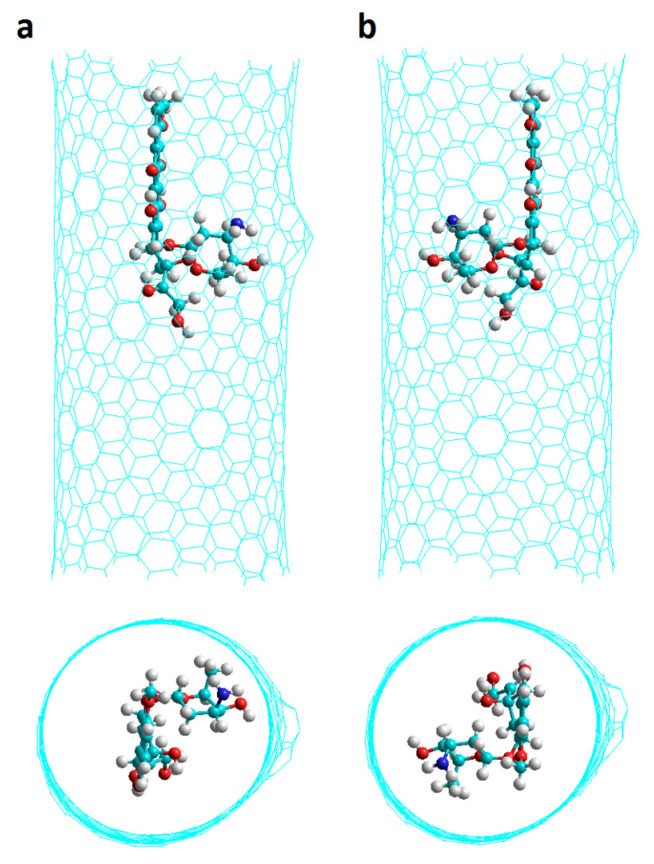
The encapsulated DOX orientations into a chiral nanotube having one haeckelite defect. (**a**) Hk1-DoxDIn.v1p; the DOX NH_2_ group is oriented in a direction proximal to the defect; (**b**) Hk1-DoxDIn.v1o; the DOX NH_2_ group is oriented in an opposite direction. Lateral and frontal views shown.

Chiral nanotubes Ch(13,08) with one Hk defect (named Hk1) and two Hk defects (named Hk2) having 0N, 4N and 8N were studied considering different initial positions of the DOX: in the region of the defect (D), in the regular region of the nanotube (R) (there are no defects in that area) with the DOX NH_2_ group pointing towards the center of the nanotube (v1 orientation) or to the inverse direction (v2 orientation) as shown in Figure 2 for Hk2 chiral nanotubes. Other additional DOX orientations refer to Hk1 nanotubes: when the DOX NH_2_ group is oriented in a direction proximal to the defect (p) or is in the direction opposite to the defect (o) as shown in Figure 3.

#### 2.1.1. Chiral Nanotubes with HK1 Defects

**Figure 4:**
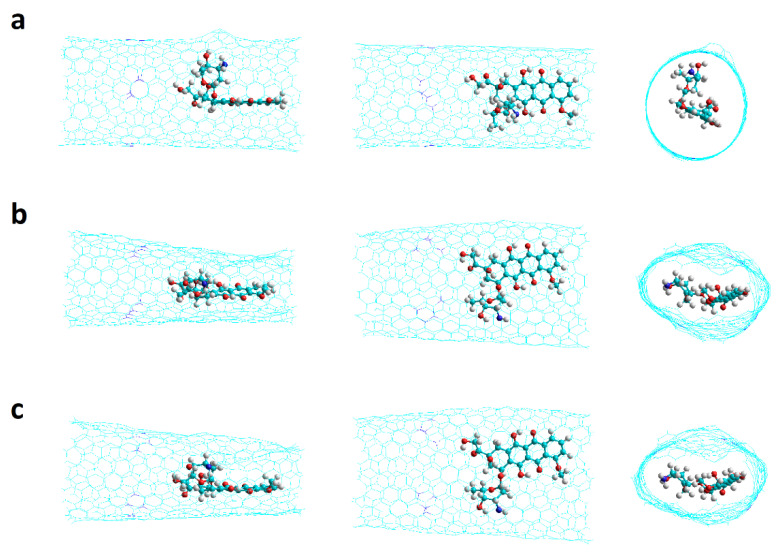
Representation of encapsulated DOX-CNT complex for nitrogen-doped chiral nanotube with one haeckelite defect, Ch(13,08)8N-Hk1-DoxDIn.v1p, at different simulation times. (**a**) 0 ns; (**b**) 2 ns; (**c**) 100 ns. Two laterals and one frontal view are shown.

For chiral nanotubes with Hk1 defects (Hk1 chiral nanotubes), the results predict a similar behavior for both undoped, 0N, and nitrogen doped nanotubes having 4N and 8N. In all three cases, the DOX-CNT interaction is favored when the DOX is located in the defect area with the NH_2_ group pointing towards the center of the nanotube (v1 orientation) in the proximal direction close to the defect as shown in Table 1 (runs 2, 10 and 18 for 0N, 4N and 8N, respectively) with Poisson–Boltzman (PB) binding energies of −102, −99 and −102 kcal/mol, respectively. Coherently, most of these systems exhibit equilibrium distances with values between 3.2 and 3.6 Å evidencing stronger DOX-CNT interactions which are favored by the orientation of the DOX that facilitates the NH-π interaction. In Figure 4 the initial conformation of the Ch(13,08)8N-Hk1-DoxDIn.v1p complex is shown together with the final conformations after 2 ns of MD simulation and after 100 ns. It is observed that DOX does not move towards the regular part of the nanotube but interacts with the defect and as a result, in that area, there is a significant deformation of the nanotube. These results obtained for chiral nanotubes show the same behavior as was reported for armchair nanotubes with more favorable DOX-CNT interactions for systems in which DOX is located in the defect region and when it is oriented with its nitrogen atom directed towards the center of the nanotube. However, armchair nanotubes having one haeckelite defect exhibit DOX-CNT binding energies that are more exothermic suggesting stronger DOX-CNT interactions [33].

DOX-CNT systems, doped with 4N and containing Hk1 defect exhibit quite similar DOX-CNT PB binding energy values between −79 and −77 kcal/mol, probably accounting for an electronic distribution that interacts with the drug in a similar way regardless of DOX position and orientation. This could be due to the arrangement of nitrogen atoms which are part of two pyrimidine rings placed opposite each other on the walls of the nanotube. 

#### 2.1.2. Chiral Nanotubes with Hk2 Defects

**Figure 5:**
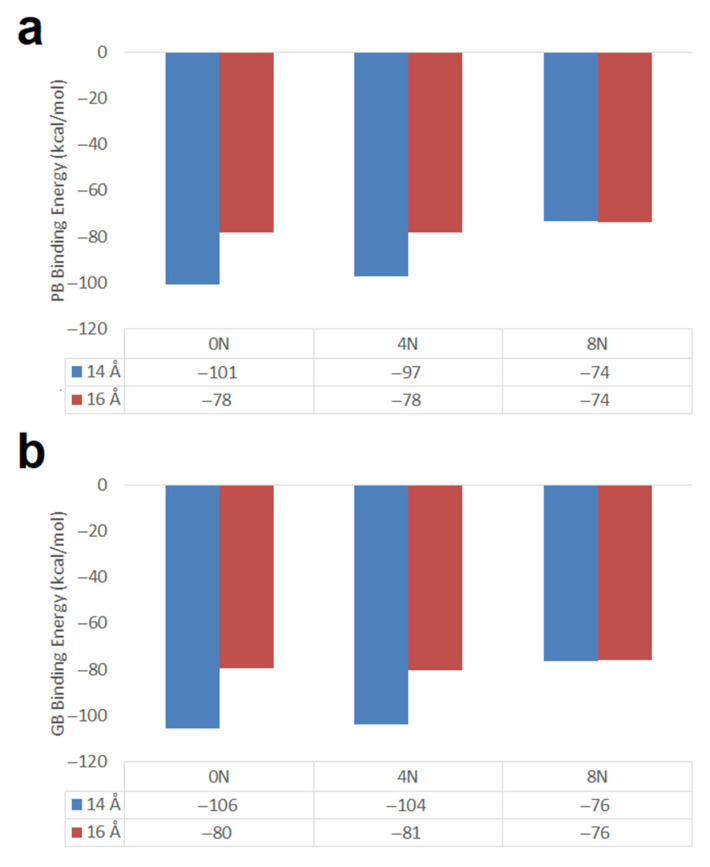
DOX-CNT binding energies for nitrogen-doped and undoped chiral nanotubes of 19 Å length with different diameter, having Hk2 defects and calculated with RESP charges for DOX. (**a**) PB binding energies; (**b**) GB binding energies. Blue is for 14 Å diameter and red is for 16 Å diameter.

Carbon nanotube diameter effect. The nitrogen doped and undoped Hk2 chiral (13,08) CNTs of 14 Å diameter and 19 Å length showed better DOX-CNT PB and GB binding energies than the corresponding Hk2 chiral (13,10) CNTs of 16 Å diameter (calculated with RESP charges for DOX) as shown in Figure 5. This was an expected result considering PM6-DH2 and M06-2X theoretical calculations in the scheme of ONIOM for the DOX encapsulation in armchair CNTs without defects [20], and also molecular dynamics studies on armchair, zigzag and chiral nanotubes with reported values of 14 Å as an optimal value of the nanotube diameter for encapsulating the DOX [32]. A diameter of 14 Å allows the proper curvature of the nanotube for the formation of different attractive and complementary non-covalent interactions between the nanotube and the DOX that stabilize the entire system which is also fulfilled in this case of nanotubes containing two haeckelite defects in their structure.

Hk2 chiral nanotubes of diameter 14 Å exhibit less favorable DOX-CNT PB binding energies for nitrogen doped nanotubes in the order 0N > 4N > 8N with values of −101, −97 and −74 kcal/mol, respectively (see Figure 5), which predicts stronger DOX-CNT interactions for undoped Hk2 chiral (13,08) nanotubes. These values were calculated using RESP charges for DOX. RESP (restrained electrostatic potential) approach to derive partial charges has been reported as having a lower average error than MM3 and CHARMm in a study considering 55 molecules [45]. Mean SD for the PB binding energy values between 2.6 and 3.2 kcal/mol and between 2.6 and 3.1 kcal/mol for the GB binding energy values were observed, being in all cases less than 4.2%.

Carbon nanotube length and DOX pose effects. Longer Hk2 chiral nanotubes (34 Å length) exhibit more exothermic DOX-CNT PB binding energy values than Hk2 chiral nanotubes of 19 Å length, for both nitrogen doped and undoped nanotubes. For the undoped nanotubes (0N) and those doped with 4N, a clear preference of the DOX for the nanotube defect zone is shown with DOX-CNT PB binding energy values of −109 and −104 kcal/mol, respectively (runs 1 and 5, Table 2). When the DOX is in the regular zone of the nanotube, no significant differences are observed between the v1 or v2 orientations of the DOX. However, in cases where the DOX is initially located in the defect zone, stronger interactions are predicted for v1 DOX orientation for both undoped and 4N doped systems as shown in Table 2 (runs 1 vs. 2 and 5 vs. 6).

**Figure 6:**
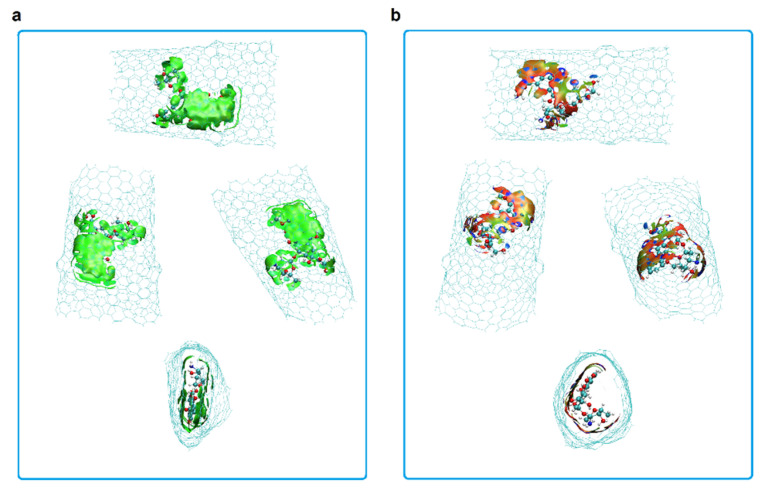
Representation of non-covalent interactions (NCI) for DOX encapsulation inside chiral carbon nanotubes having two haeckelite defects. (**a**) DOX D position; (**b**) DOX R position. Cutplot 0.01 0.1. Blue surfaces indicate strong interactions; green means weak interactions; red means repulsion [47]. Different comparative lateral and frontal views shown.

The best system for DOX encapsulation in Hk2 chiral nanotubes is therefore Ch(13,08)0N-HK2-DoxDIn.v1 (run 1, Table 2) with the DOX in the defect region and v1 orientation (with the nitrogen pointing towards the center of the nanotube). In this conformation, the formation of the non-covalent DOX-CNT interactions is facilitated, which are mainly constituted by π–π stacking interactions complemented by NH-π, CH-π, C=O-π and van der Waals interactions [20,31,46]. Figure 6 shows the non-covalent interactions for the most favorable case with the DOX in the area of the nanotube defect (a large green surface is observed) and as a comparison, the same nanotube with the DOX encapsulated in the regular area (less green regions and more red regions are observed), with PB DOX-CNT binding energy values of −109 and −80 kcal/mol, respectively (runs 1 and 3, Table 2). A program specially developed for the visualization of non-covalent interactions (NCI) was used [47].

Under similar MD simulation conditions but considering RESP charges for DOX, Hk2 chiral (13,08) nanotubes (−101 kcal/mol, Figure 5) predict stronger DOX-CNT interactions than reported Hk2 armchair (10,10) nanotube (−83.4 kcal/mol) with the encapsulated DOX located in the defect zone in v1 orientation [33]. Hk2 chiral nanotubes predict stronger DOX-CNT interactions than reported Hk2 zigzag (18,0) nanotubes which exhibit values of DOX-CNT PB binding energies of −78.7 kcal/mol for undoped nanotubes [33]. The three types of CNTs in comparison have diameters of 14 Å. In this way, in terms of chirality and according to the indicated results, Hk2 nanotubes exhibit the following order of ability to encapsulate the DOX: chiral > armchair > zigzag, despite chiral nanotubes are shorter than zigzag and armchair nanotubes (19 vs. 34 Å length). The enhanced ability of chiral nanotubes with respect to other nanotubes to encapsulate DOX was reported also for perfect CNTs through MD simulation studies considering RESP charges for DOX [32].

### 2.2. Nanotubes with Stone–Wales Defects

**Figure 7:**
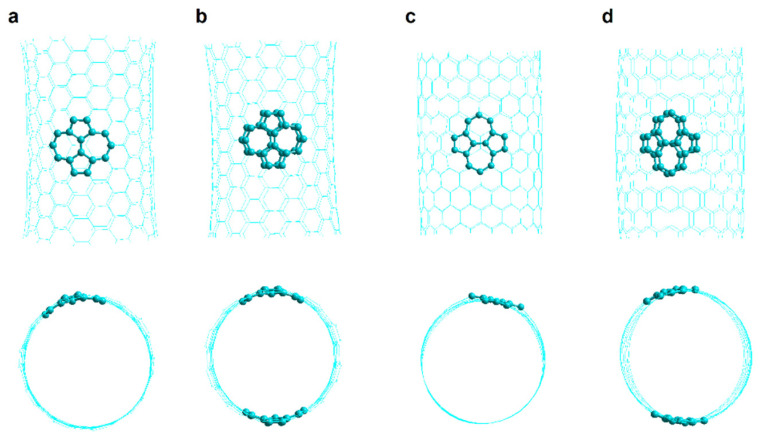
Representation of carbon nanotubes having one and two Stone–Wales defects. (**a**) SW1 armchair; (**b**) SW2 armchair; (**c**) SW1 zigzag; (**d**) SW2 zigzag. Side and front views shown.

The encapsulation of the DOX was studied in armchair and zigzag nanotubes that have one and two Stone–Wales defects (SW1 and SW2, respectively) as shown in Figure 7.

#### 2.2.1. SW1 and SW2 Armchair Nanotubes 

**Figure 8:**
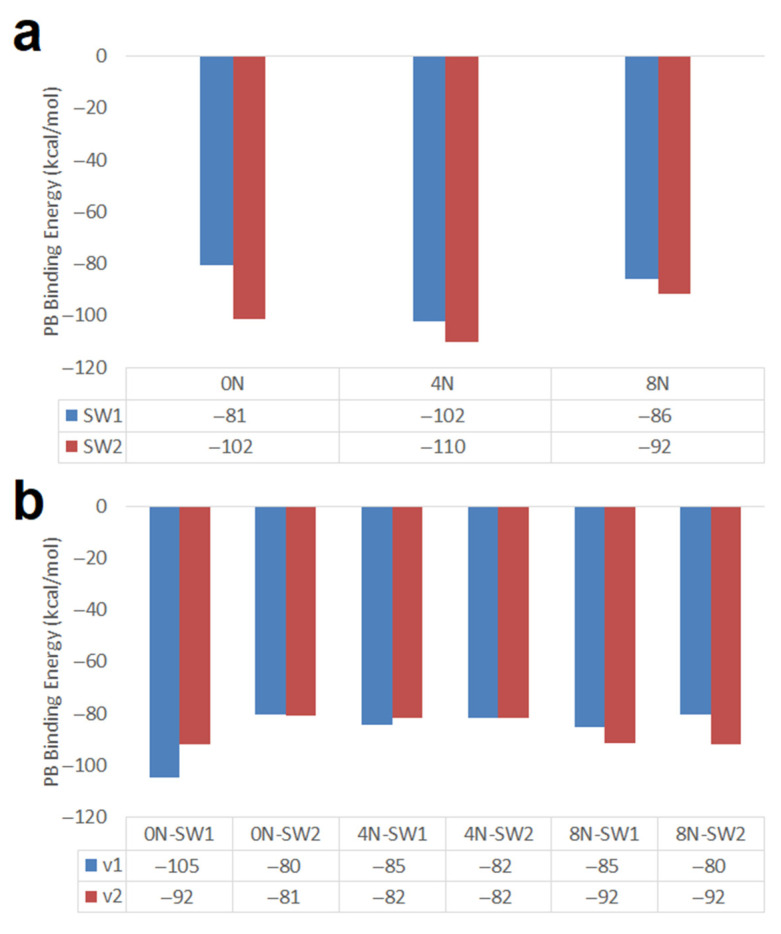
DOX-CNT PB binding energies for nitrogen-doped and undoped armchair (10,10) nanotubes of different length having one and two Stone–Wales defects. (**a**) 20 Å length; (**b**) 34 Å length.

Armchair (10,10) nanotubes of 20 Å and 34 Å in length having Stone–Wales defects were studied, which showed different behaviors in DOX encapsulation. The shorter nanotubes (20 Å length) exhibit a significant stronger interaction with the DOX in two situations: (i) when they are of the SW2 type (with two defects, doped and undoped) in comparison with SW1 nanotubes and (ii) when they are doped with 4N (SW1 and SW2) as is clearly depicted in Figure 8a. Nitrogen doped SW1 and SW2 armchair (10,10) nanotubes of 20 Å length predict stronger interactions with the DOX than corresponding longer SW1 and SW2 nanotubes of 34 Å in length as shown in Figure 8 particularly for 4N doped nanotubes. 

In contrast, longer SW1 armchair nanotubes predict somewhat stronger interactions than longer SW2 armchair nanotubes the difference being more significant for the undoped SW1 and SW2 nanotubes with PB DOX-CNT binding energy values of −105 and −80 kcal/mol for, respectively, with DOX v1 orientation, and for DOX v2 orientation −92 vs. −81 kcal/mol, respectively, as shown in Figure 8b.

**Figure 9:**
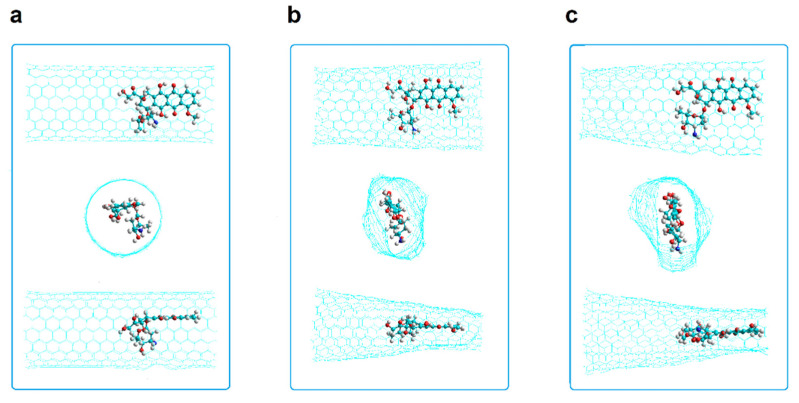
Representation of encapsulated DOX-CNT complex for undoped *armchair* nanotube with one Stone–Wales defect, A(10,10)0N-SW1-DoxDIn.v1 at different molecular dynamics simulation times. (**a**) 0 ns; (**b**) 2 ns; (**c**) 74 ns. Two lateral and frontal views shown.

The most exothermic PB DOX-CNT binding energy with a value of −110 kcal/mol correspond to the shorter 4N-doped SW2 armchair nanotube which predicts the stronger DOX-CNT interactions. In the shorter SW2 armchair nanotubes the DOX is symmetrically located and can interact with both of the two defects which favors DOX-CNT interactions. Meanwhile for shorter SW1 armchair nanotubes the DOX interacts with just one defect only. In contrast, the less exothermic PB DOX-CNT binding energies correspond to longer SW1 and SW2 armchair nanotubes with values between −92 and −80 kcal/mol. The only exception is the longer SW1 armchair nanotube where the DOX is in the v1 orientation showing a PB binding energy of −105 kcal/mol. Figure S1 (Supplementary Materials) clearly shows the differences in relative DOX-CNT binding energies. For short nanotubes (20 Å long), SW2 exhibits more exothermic DOX-CNT binding energies than SW1, with 4N doping predicting the strongest DOX-CNT interactions. Within the long nanotubes (34 Å long) the most exothermic DOX-CNT binding energies correspond to the undoped SW1 nanotubes with v1 orientation. In Figure 9 its initial structure is shown and also at 2 ns and 74 ns of simulation where a probable double π–π interaction of the DOX with the two opposite walls of the nanotube is appreciated which generates a significant deformation of the nanotube in addition to its NH-π interaction with the DOX amino group that helps stabilize the system. In the longer nanotubes, the DOX interacts with the regular part of the nanotube also. Apparently the interactions DOX-Stone–Wales defects are stronger than the interactions DOX-regular CNTs. 

#### 2.2.2. SW1 and SW2 Zigzag Nanotubes

**Figure 10:**
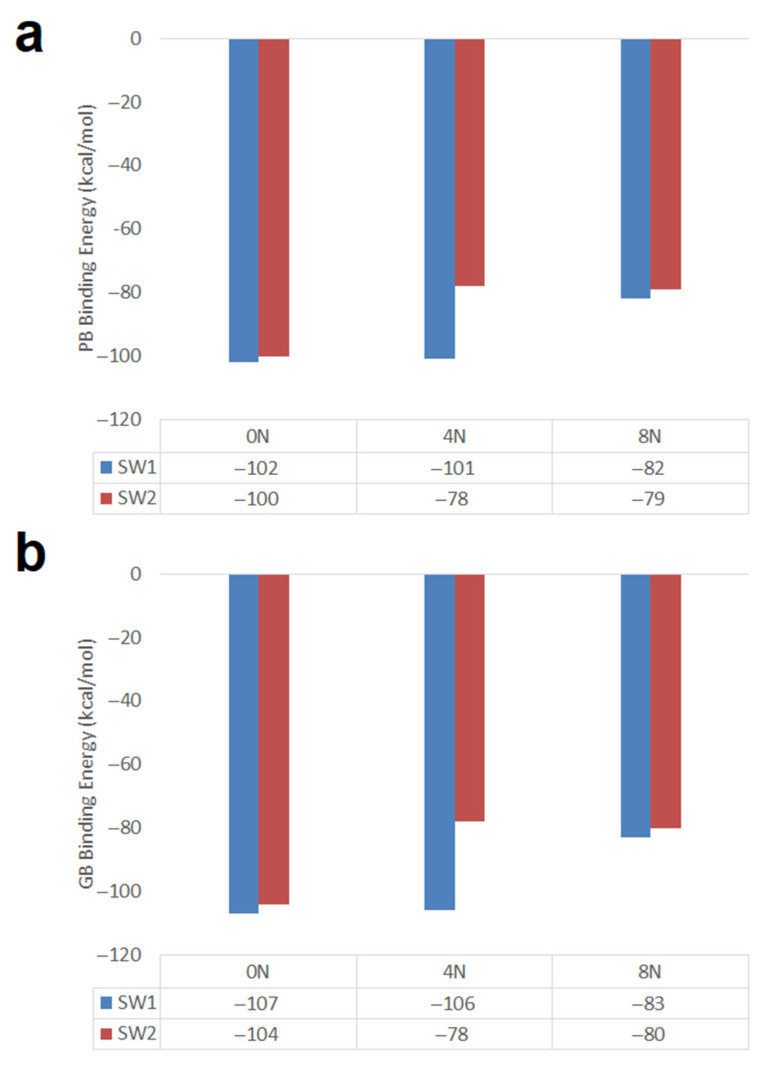
Representation of the PB and GB DOX-CNT binding energies for nitrogen-doped and undoped zigzag (18,0) nanotubes having one and two Stone–Wales defects. (**a**) PB binding energy; (**b**) GB binding energy.

Zigzag (18,0) nanotubes of 20 Å in length and 14 Å diameter having one and two Stone–Wales defects were studied. In Figure 10 the PB and GB DOX-CNT binding energies are shown for undoped nanotubes and for nanotubes doped with 4 and 8 nitrogen atoms. It is observed that both types of binding energies show the same tendency (as was also observed in Figure 5 for chiral nanotubes) and that undoped zigzag nanotubes predict stronger DOX-CNT interactions than doped ones, both with one or two SW defects.

Zigzag nanotubes having one SW defect (SW1) exhibit stronger DOX-CNT interactions than those having two SW defects (SW2). This behavior is more significant for nanotubes doped with 4 nitrogen atoms. Structurally, the presence of 4N means that there are two pyrimidine rings in the nanotube. Zigzag nanotubes with a SW1 defect doped with 8 nitrogen atoms (have four pyrimidine rings in the nanotube) show weaker DOX-CNT interactions than in the case of nanotubes doped with 4N. Undoped SW1 and SW2 nanotubes show similar DOX-CNT PB binding energies of −102 and −100 kcal/mol, respectively. Apparently DOX accommodates better in a space with SW defects but free from the influence of the nitrogen electron cloud. No great difference is observed between the DOX-CNT binding energies obtained for undoped SW1 and SW2 nanotubes and those for SW1 nanotubes doped with 4N with a PB binding energy of −101 kcal/mol; but for 4N-doped SW2 nanotubes, the DOX-CNT PB binding energy decreases significantly to −78 kcal/mol (probably DOX is prevented from accommodating in a narrower space). The same effect is observed when SW1 zigzag nanotubes doped with 4N are compared with those doped with 8N (−101 kcal/mol vs. −82 kcal/mol). Mean SD for the PB binding energy values ranged between 2.7 and 4.0 kcal/mol and between 2.9 and 4.1 kcal/mol for the PB and GB binding energy values, respectively, being in all cases less than 4%. Figure S2 (Supplementary Materials) clearly shows the DOX-CNT relative binding energies with respect to the non-doped nanotubes. SW1 nanotubes have more exothermic DOX-CNT binding energies than SW2. Furthermore, the non-doped SW1 nanotubes exhibit DOX-CNT binding energy values not very distant from the 4N-doped nanotubes, being the 8N-doped nanotubes the ones with the least favorable binding energies.

The equilibrium distances d_p-NT_ between the DOX anthraquinonic planar part and the nanotube wall ranged between 3.3 and 3.7 Å and the equilibrium distances d′_p-NT_ between the same DOX planar part and the nanotube wall in the opposite direction it ranged between 3.8 and 3.9 Å for cases having DOX-CNT PB binding energy between −100 and −102 kcal/mol. On the other hand, for cases with PB binding energy between −78 and −82 kcal/mol, the distances d′_p-NT_ ranged between 7.6 and 8.2 Å. So, for binding energies that predict strongest DOX-CNT interactions, a deformation of the nanotube is observed, which can also be seen in Figure 4 and Figure 9 for chiral and armchair nanotubes, respectively, probably due principally to a double π−π stacking between the DOX anthraquinonic planar rings and both of the opposite CNT walls, and DOX-CNT van der Waals interactions. The equilibrium d_N-NT_ distances between the DOX nitrogen atom and the nanotube inner wall exhibit values between 3.3 and 4.0 Å regardless of the value of the binding energies.

## 3. Discussion

It is interesting to find out how the structural parameters of nanotubes affect the relative DOX-CNT binding energies which could allow us to infer about the DOX-CNT interactions for nanotubes of different types. The aim is to predict nanotube structures that can develop more exothermic binding energies; that is, that produce more favorable DOX-CNT interactions, without fear that the desorption of the drug be difficult since the acidic pH existing in the tumor environment facilitates protonation and release of the drug, which has been verified in different systems [14,15,16,17,38].

In the present work, it was found that a diameter of 14 Å favors the DOX-CNT interaction for Hk2 chiral nanotubes, in agreement with works reported for other defect-free nanotubes [20,32]. Furthermore, it was found that the strength of the DOX-CNT interaction decreases as the number of doping nitrogen atoms increases (0N > 4N > 8N). This relative trend was found both for long Hk2 chiral nanotubes (33 Å length) using Mulliken charges for DOX, as well as for short Hk2 chiral nanotubes (19 Å length) using RESP charges for DOX. However, using similar simulation conditions, Hk1 and Hk2 armchair and zigzag nanotubes (33 Å length) showed that nitrogen-doped systems had more favorable binding energies than non-doped ones [33]. Chirality shows to be an important parameter that controls the effect of the presence of nitrogen in the nanotube, modifying the distribution of electron density and therefore the DOX-CNT interactions in such a way that the presence of nitrogen in the chiral nanotubes destabilizes the association DOX-CNT unlike of what happens in armchair and zigzag nanotubes.

The initial poses of the DOX in the CNT proved to be important, finding better interactions for chiral nanotubes when the DOX is located in the defect part, oriented in such a way that its nitrogen atom points towards the center of the nanotube and is close to the defect. Apparently, this conformation favors π–π, van der Waals interactions and also electrostatic interactions of the NH-π, OH-π, C=O-π type as was observed in Figure 6. These DOX-CNT interactions in the cases of more electronegative binding energies translate into a significant deformation of the nanotube, as observed in Figure 4 and Figure 9 with PB binding energies of −102 and −105 kcal/mol, for chiral and armchair nanotubes, respectively, which could be explained by the π–π interaction of the flat anthraquinonic system of the DOX with both opposite walls of the nanotube. The deformation of the nanotube has been observed in other MD simulation works [32,33], and also in works carried out using the PM6-DH2 and M06-2X methods [20].

On the other hand, the short SW2 armchair nanotubes (20 Å length) showed better DOX-CNT binding energies than the long SW2 armchair nanotubes (34 Å length). Although the short SW2 armchair nanotubes showed more exothermic DOX-CNT binding energies than the respective short SW1 armchair nanotubes, this trend did not hold for the long armchair nanotubes. 

In contrast, the short SW1 zigzag nanotubes showed more exothermic DOX-CNT binding energies than the short SW2 zigzag nanotubes. For the SW1 and SW2 zigzag nanotubes it was found that the strength of the DOX-CNT interactions decreased in the presence of nitrogen in the order 0N > 4N > 8N, in a similar way to that shown by the Hk2 chiral nanotubes. Nitrogen-doped SW1 armchair and zigzag nanotubes showed stronger DOX-CNT interactions than the respective Hk2 chiral nanotubes.

There is a lack of experimental studies on the determination of DOX-CNT binding energies. Only one estimation is known in an aqueous system, of about 11.5 kcal/mol for DOX-CNT complexes 100 nm long and 2 to 3 nm in diameter [19]. However, the formation of stable DOX-CNT conjugates has been determined experimentally, verified by atomic force microscopy (AFM) and scanning tunneling microscopy (STM) images, using single-walled CNTs (SWCNTs) with diameter 1–1.5 nm and a few hundred nanometers long, purchased from ILJIN Co., Inc., Korea. [7]. In none of these cases is the chirality of the nanotube or the presence of defects specified. 

It is not easy to confront theoretical studies that depend on a variety of factors, such as, in the field of molecular dynamics, the assignment of the type of atom involved in the consideration of the bonded and non-bonded parameters, or the definition of dihedrals and other interpretations of the description of the force field used [48].

The method used in the present work is very useful to obtain the DOX-CNT bonding energy values since it is comparatively fast and reproducible. Its calculation is based on the MM/PBSA and MM/GBSA approaches starting from the equilibrium energy values obtained through molecular dynamics simulation. Although the calculation does not consider the translational entropy (huge computational cost), it has been shown that it does provide adequate values of the relative binding energies, which have been experimentally validated in biological systems [49,50] and it has recently been reported that when the MM/PBSA and MM/GBSA are used together with empirical corrections, they allow better experimental correlations [51]. 

DOX-CNT binding energies for the encapsulation of DOX on the walls of armchair nanotubes without defects at the PM6-DH2 level, in aqueous solution, showed values between −51.6 and −53.7 kcal/mol as a function of diameter [20].

As a reference, the nitrosamine adsorption free energy on open-ended Stone–Wales defective (5,5) armchair nanotube calculated by DFT methods at the B3LYP/6-31G(d) level of theory showed a value of −137.14 kcal/mol [52]. DOX-CNT binding free energy values obtained by the MM/PBSA and MM/GBSA methods for (10,10) armchair nanotubes without defects were −43 kcal/mol for DOX adsorption and fluctuated between −109 and −90 kcal/mol for DOX encapsulation [32]. For (10,10) armchair nanotubes with bumpy defects they ranged between −96 and −83 kcal/mol. (10,10) armchair nanotubes with haeckelite defects ranged from −104 to −80 kcal/mol depending on the number of defects and the presence of nitrogen [33]. 

Interestingly, although the DOX-CNT binding free energy values previously reported by us using the current methodology [32], are far from the values calculated by Wang and Xu [20] by means of PM6-DH2 and M06-2X methods, in both works, after a systematic study, it was found that the best nanotube diameter for encapsulate the DOX is 14 Å. This fact, together with the deformation of the nanotube observed with both methods means that the behavior and ability ranking of the nanotubes to associate with DOX coincides in both works, so it could be considered as an indirect validation of the use of the MM/PBSA and MM/GBSA methods to predict the DOX-CNT binding free energies for different nanotube structures. 

The binding energy calculations are very useful to study molecular interactions, complex stability, getting information necessary for drug design, carrier design, inhibitor design, etc. The methods that allow obtaining the most accurate values are expensive in computational time and resources. Semi-empirical methods have been found to allow a good level of accuracy when compared to experimental measurements, and to be relatively fast and of low computational cost. The prediction of binding energies through the MM/PBSA approach increases in efficiency and accuracy when using the partial charges of the ligand determined by semi-empirical methods. A study with 50 protein-ligand systems revealed an excellent performance of the PM7 and AM1 systems, comparable to B3LYP which requires a huge computational cost, reaching the conclusion that the semi-empirical methods AM1 or PM7 provide partial charges of the proteins that help to improve the prediction of binding energies through methods such as solvated interaction energy (SIE) and MM/PBSA in a highly efficient and accurate way [53]. These results validate in some way the use of AM1 in this work.

To summarize, the structural parameters that dominate the behavior of defective nanotubes related to DOX encapsulation are interdependent and in order of importance, from what is observed in this work, the following can be mentioned: chirality, diameter and length of the nanotube; presence of nitrogen atoms as dopants; number of defects present and type of defects. Additionally, the initial pose of the DOX in the nanotube also affects the intermolecular attractive forces. We have found that the initial pose of DOX affects the DOX-CNT binding energies and we have been able to clarify that DOX-CNT interactions are favored when the DOX amino group points towards the center of the nanotube with an orientation close to the defects. It would be interesting in a next stage to do a simulation that considers different conformations of DOX.

As future work, we are doing research on chiral nanotubes with Stone–Wales defects. However, based on the known simulation studies, which are not directly comparable, we believe a global systematic approach is needed. This new comparable approach should consider the best values reported for each system (and also some of the values found as unfavorable, for comparison) to perform the new simulations. To have a valid ranking of CNTs activity, as drug carriers, with or without defects, at least two conditions should be considered for the new simulations: (i) comparable structural parameters (for example, same diameter, length and number of defects, including nitrogen); and (ii) comparable simulation conditions (same version of the package of programs, same force field, type of charges, type of solvation, among others). Due to the complexity and the high number of variables, we believe that the use of Artificial Intelligence tools will be very useful to improve the study of the design of new drug carriers and also for other related applications. Anyway, refinement of the results for the best systems found could be done using simulation methods and conditions that guarantee greater accuracy.

## 4. Simulation Methods

The nanotubes were prepared as single-walled open nanotubes finished in hydrogen with the help of HyperTube [54] and Hyperchem [55] and optimized to the Austin Model One (AM1) level. Chiral CNTs with one and two haeckelite defects (Hk1 and Hk2, respectively) and also armchair and zigzag CNTs with one and two Stone–Wales defects (SW1 and SW2, respectively) were prepared as shown in Figure 7. Molecular dynamics simulations were performed with AMBER16 [56,57]. A program made at home was use for the preparation of the necessary files and instructions to run the MD program [32]. The combined GAFF and ff99SB force fields were used. The DOX was optimized at the level of HF/6-31G* for some specific cases, just to get the restrained electrostatic potential (RESP) partial charges by using the antechamber AMBER program [45,58]. For the rest of the structures the AM1-Mulliken charges were used. All the DOX-CNT complexes were neutral systems, solvated in an explicit solvent, in a 10 Å octahedron water box using bondi radii under periodic boundary conditions. TIP3P was used as water model.

Simulations were run after some steps of minimization (1000 stages) and heating (from 0 to 300 K), both at constant volume, density balance (50 ps) and equilibrium (500 ps) both of them at constant pressure. Then, the production step consisting of six independent short stages (250 ps each) at constant pressure was carried out. This procedure of using several independent short simulations instead of a single long simulation has being recognized as efficient and accurate [50,59]. In effect, as it is shown in Figure 4, a simulation performed for 100 ns (using several short simulation) revealed similar energy and geometry results than the simulation procedure with short stages as it was indicated above. The drug-nanotube binding free energies were determined through the MM/PBSA and MM/GBSA methods implemented in AMBER [56]. These methods were applied on an ensemble of 200 uncorrelated snapshots collected from the equilibrated molecular dynamics simulation for calculating the Gibbs free energy difference of the solvated bound (G_CNT-DOX_) and unbound states of the drug (G_DOX_) and nanotube (G_CNT_) molecules Equation (1).

ΔG = G_CNT-DOX_ − G_CNT_ − G_DOX_ (1)

Each of these terms were obtained according to Equation (2):

(2) $$G=E_{vdW}+E_{bond}+E_{el}+E_{pol}+E_{np}-TS$$

where *E_vdW_* (van der Waals), *E_bond_* (bond, angle, dihedral) and *E_el_* (electrostatic) are the standard molecular mechanics (MM) energy terms; *E_pol_* (polar term) is calculated by solving the Poisson–Boltzmann (PB) or the generalized born (GB) equation; *E_np_* (non-polar term) is estimated from a linear relation with the solvent accessible surface area (SA); *T* is the absolute temperature and *S* is the entropy term (estimated through a normal-mode analysis of the vibrational frequencies). The fact that the binding free energies are calculated without considering the translational entropy must be borne in mind when interpreting the results. To obtain a good ranking of nanotube ability for encapsulating DOX based on the DOX-CNT binding energy values this is a convenient method in terms of computational resources, but not for obtaining absolute DOX-CNT binding energy values.

## 5. Conclusions

The DOX-CNT binding energies calculated in this work allow us to establish interesting trends such as:

- When using RESP charges, DOX encapsulation inside Hk2 nanotubes of 14 Å diameter, predicts that the DOX-CNT attractive forces decrease in the following order of nanotube chirality: chiral > armchair > zigzag in agreement with that reported in a MD study under similar conditions, for defect-free nanotubes.
- Hk2 chiral nanotubes (short and long) favor DOX-CNT interactions which decrease in the presence of nitrogen as a dopant in the order 0N > 4N > 8N.
- Short armchair nanotubes promote more favorable DOX-CNT interactions if they are SW2; but long armchair nanotubes favor the DOX-CNT interactions if they are SW1. When the DOX is located close to the defect, a better interaction occurs than when it is located in the defect-free zone of the nanotube. The interaction is also improved if the DOX nitrogen atom is close to the defect.
- Short SW1 zigzag nanotubes favor DOX-CNT interactions which decrease in the presence of nitrogen as a dopant in the order 0N > 4N > 8N, like the Hk2 chiral nanotubes. SW1 zigzag nanotubes exhibit more exothermic DOX-CNT binding energies than SW2 zigzag nanotubes. In both cases, the undoped SW defective zigzag nanotubes are predicted to exhibit stronger DOX-CNT interactions than corresponding nitrogen-doped nanotubes.

## Figures and Tables

**Table 1:** DOX-CNT Poisson–Boltzman (PB) and generalized bond (GB) binding energies (in kcal/mol) for the nitrogen doped and undoped chiral nanotubes (34 Å length) having one haeckelite defect, Hk1, considering encapsulated system. d_p-NT_ are the distances from the DOX anthraquinonic plane to the nanotube wall; d_N-NT_ is the distance from the DOX nitrogen atom to the nanotube wall. All distances are expressed in Å.

Run	Type ^1^	PB	GB	d_p-NT_	d_N-NT_
1	Ch(13,08)0N-HK1-DoxDIn.v1o	−78	−77	3.7	3.7
2	Ch(13,08)0N-HK1-DoxDIn.v1p	−102	−104	3.6	3.5
3	Ch(13,08)0N-HK1-DoxDIn.v2o	−79	−80	3.6	3.9
4	Ch(13,08)0N-HK1-DoxDIn.v2p	−85	−87	3.8	3.6
5	Ch(13,08)0N-HK1-DoxRIn.v1o	−78	−77	3.4	3.1
6	Ch(13,08)0N-HK1-DoxRIn.v1p	−80	−80	3.4	3.6
7	Ch(13,08)0N-HK1-DoxRIn.v2o	−79	−78	3.8	4.9
8	Ch(13,08)0N-HK1-DoxRIn.v2p	−80	−80	3.5	3.2
9	Ch(13,08)4N-HK1-DoxDIn.v1o	−77	−76	3.5	3.4
10	Ch(13,08)4N-HK1-DoxDIn.v1p	−99	−102	3.4	3.3
11	Ch(13,08)4N-HK1-DoxDIn.v2o	−78	−80	3.8	3.9
12	Ch(13,08)4N-HK1-DoxDIn.v2p	−79	−80	3.4	3.0
13	Ch(13,08)4N-HK1-DoxRIn.v1o	−77	−77	3.6	4.0
14	Ch(13,08)4N-HK1-DoxRIn.v1p	−79	−81	3.6	3.5
15	Ch(13,08)4N-HK1-DoxRIn.v2o	−78	−79	4.2	4.6
16	Ch(13,08)4N-HK1-DoxRIn.v2p	−79	−80	3.5	3.2
17	Ch(13,08)8N-HK1-DoxDIn.v1o	−78	−79	3.6	3.5
18	Ch(13,08)8N-HK1-DoxDIn.v1p	−102	−104	3.4	3.2
19	Ch(13,08)8N-HK1-DoxDIn.v2o	−78	−79	3.8	3.6
20	Ch(13,08)8N-HK1-DoxDIn.v2p	−78	−79	3.4	3.4
21	Ch(13,08)8N-HK1-DoxRIn.v1o	−79	−80	3.4	3.4
22	Ch(13,08)8N-HK1-DoxRIn.v1p	−81	−81	3.6	3.4
23	Ch(13,08)8N-HK1-DoxRIn.v2o	−73	−76	3.5	3.2
24	Ch(13,08)8N-HK1-DoxRIn.v2p	−81	−82	3.5	3.1

^1^ DoxD means DOX position is in the defect zone; DoxR is for the DOX in the regular part of the nanotube; v1 means the nitrogen atom of the DOX is oriented towards the center of the tube; v2 indicates the inverse orientation; v1p indicates that the DOX nitrogen atom is located in a proximal space regarding the defect meanwhile v1o is used to indicate that the DOX nitrogen atom is located in an opposite space regarding the defect as shown in Figure 3.

**Table 2:** Poisson–Boltzman (PB) and generalized bond (GB) binding energies (in kcal/mol) for the nitrogen doped and undoped chiral nanotubes (34 Å length) having two haeckelite defects, Hk2, considering encapsulated system. d_p-NT_ are the distances from the DOX anthraquinonic plane to the nanotube wall; d_N-NT_ is the distance from the DOX nitrogen atom to the nanotube wall. All distances are expressed in Å.

Run	Type ^1^	PB	GB	d_p-NT_	d_N-NT_
1	Ch(13,08)0N-HK2-DoxDin.v1	−109	−112	3.4	3.4
2	Ch(13,08)0N-HK2-DoxDin.v2	−82	−83	3.7	3.4
3	Ch(13,08)0N-HK2-DoxRin.v1	−80	−79	3.3	3.4
4	Ch(13,08)0N-HK2-DoxRin.v2	−79	−79	3.2	4.8
5	Ch(13,08)4N-HK2-DoxDin.v1	−104	−108	3.5	4.3
6	Ch(13,08)4N-HK2-DoxDin.v2	−82	−83	3.8	3.5
7	Ch(13,08)4N-HK2-DoxRin.v1	−79	−78	4.1	3.4
8	Ch(13,08)4N-HK2-DoxRin.v2	−80	−80	3.3	3.5
9	Ch(13,08)8N-HK2-DoxDin.v1	−80	−78	3.4	3.3
10	Ch(13,08)8N-HK2-DoxDin.v2	−87	−89	3.6	3.2
11	Ch(13,08)8N-HK2-DoxRin.v1	−80	−80	3.6	3.3
12	Ch(13,08)8N-HK2-DoxRin.v2	−80	−79	3.8	3.7

^1^ DoxD means DOX position is in the defect zone; DoxR is for the DOX in the regular part of the nanotube; v1 means the nitrogen atom of the DOX is pointing towards the center of the tube; v2 indicates the inverse orientation.

## Data Availability

Does not apply.

## Supplementary Materials

The following are available online, Figure S1: DOX-CNT PB relative binding energies for nitrogen-doped and undoped (10,10) armchair nanotubes of different length having one and two Stone–Wales defects. (a) 20 Å length; reference: 4N doped SW2 nanotube with −110 kcal/mol PB DOX-CNT binding energy; (b) 34 Å length; reference: 0N SW1 nanotube with −105 kcal/mol PB DOX-CNT binding energy. Figure S2: Representation of the PB and GB DOX-CNT relative binding energies for nitrogen-doped and undoped (18,0) zigzag nanotubes having one and two Stone–Wales defects. (a) PB binding energy. Reference: 0N SW1 nanotube with −102 kcal/mol PB DOX-CNT binding energy; (b) GB binding energy. Reference: 0N SW1 nanotube with −107 kcal/mol GB DOX-CNT binding energy.

## Sample Availability

Not available.

## References

[1] Etheridge M.L., Campbell S.A., Erdman A.G., Haynes C.L., Wolf S.M., McCullough J. (2013). The big picture on nanomedicine: The state of investigational and approved nanomedicine products. Nanomed. Nanotechnol. Biol. Med..

[2] Marketing Bureau Zydus Cadila Receives US FDA Approval for Generic Doxil Liposome Injection. http://www.pharmabiz.com/NewsDetails.aspx?aid=131053&sid=2.

[3] Patel A.G., Kaufmann S.H. (2012). How does doxorubicin work?. eLife.

[4] Denard B., Lee C., Ye J. (2012). Doxorubicin blocks proliferation of cancer cells through proteolytic activation of CREB3L1. eLife.

[5] Zhang S., Liu X., Bawa-Khalfe T., Lu L.-S., Lyu Y.L., Liu L.F., Yeh E.T.H. (2012). Identification of the molecular basis of doxorubicin-induced cardiotoxicity. Nat. Med..

[6] Ferreira L.L., Oliveira P.J., Cunha-Oliveira T. (2019). Epigenetics in Doxorubicin Cardiotoxicity.

[7] Rodríguez-Galván A., Amelines-Sarria O., Rivera M., Carreón-Castro M.D.P., Basiuk V.A. (2016). Adsorption and Self-Assembly of Anticancer Antibiotic Doxorubicin on Single-Walled Carbon Nanotubes. Nano.

[8] Lalan M., Jani D. (2021). Toxicological Aspects of Carbon Nanotubes, Fullerenes and Graphenes. Curr. Pharm. Des..

[9] Contreras  M.L., Rozas R. (2020). Carbon Nanotubes: Toxicological Properties, Use as Drug Delivery Material and Computational Contribution to Predict their Properties Including Structures with Topological Defects. Adv. Clin. Toxicol..

[10] Mamidi N. (2019). Cytotoxicity Evaluation of Carbon Nanotubes for Biomedical and Tissue Engineering Applications. Perspect. Carbon Nanotub..

[11] Ali-Boucetta H., Kostarelos K. (2013). Pharmacology of carbon nanotubes: Toxicokinetics, excretion and tissue accumulation. Adv. Drug Deliv. Rev..

[12] Nayak T., Leow P., Ee P.-L., Arockiadoss T., Ramaprabhu S., Pastorin G. (2010). Crucial Parameters Responsible for Carbon Nanotubes Toxicity. Curr. Nanosci..

[13] Venkataraman A., Amadi E.V., Chen Y., Papadopoulos C. (2019). Carbon Nanotube Assembly and Integration for Applications. Nanoscale Res. Lett..

[14] Pei B., Wang W., Dunne N., Li X. (2019). Applications of Carbon Nanotubes in Bone Tissue Regeneration and Engineering: Superiority, Concerns, Current Advancements, and Prospects. Nanomaterials.

[15] Saliev T. (2019). The Advances in Biomedical Applications of Carbon Nanotubes. C J. Carbon Res..

[16] Yan Y., Wang R., Hu Y., Sun R., Song T., Shi X., Yin S. (2018). Stacking of doxorubicin on folic acid-targeted multiwalled carbon nanotubes for in vivo chemotherapy of tumors. Drug Deliv..

[17] Le C.M.Q., Cao X.T., Kim D.W., Ban U.H., Lee S.H., Lim K.T. (2017). Preparation of poly(styrene-alt-maleic anhydride) grafted multi-walled carbon nanotubes for pH-responsive release of doxorubicin. Mol. Cryst. Liq. Cryst..

[18] Kumar D.S., Hasumura T., Nagaoka Y., Yoshida Y., Maekawa T., Jeyamohan P. (2013). Accelerated killing of cancer cells using a multifunctional single-walled carbon nanotube-based system for targeted drug delivery in combination with photothermal therapy. Int. J. Nanomed..

[19] Liu Z., Sun X., Nakayama-Ratchford N., Dai H. (2007). Supramolecular Chemistry on Water-Soluble Carbon Nanotubes for Drug Loading and Delivery. ACS Nano.

[20] Wang Y., Xu Z. (2016). Interaction mechanism of doxorubicin and SWCNT: Protonation and diameter effects on drug loading and releasing. RSC Adv..

[21] Das D. (2014). Carbon Nanotube and Graphene Nanoribbon Interconnects.

[22] Charlier J.-C. (2002). Defects in Carbon Nanotubes. Accounts Chem. Res..

[23] Crespi V.H., Cohen M.L., Rubio A. (1997). In Situ Band Gap Engineering of Carbon Nanotubes. Phys. Rev. Lett..

[24] Saito R., Fujita M., Dresselhaus G., Dresselhaus M.S. (1992). Electronic structure of chiral graphene tubules. Appl. Phys. Lett..

[25] Ebbesen T.W., Takada T. (1995). Topological and SP3 defect structures in nanotubes. Carbon.

[26] Sternberg M., Gruen D.M., Kedziora G., Horner D.A., Zapol P., Curtiss L.A., Redfern P.C. (2006). Carbon Ad-Dimer Defects in Carbon Nanotubes. Phys. Rev. Lett..

[27] Terrones H., Hernández E., Grobert N., Charlier J.-C., Ajayan P.M. (2000). New Metallic Allotropes of Planar and Tubular Carbon. Phys. Rev. Lett..

[28] Stone A.J., Wales D.J. (1986). Theoretical studies of icosahedral C60 and some related species. Chem. Phys. Lett..

[29] Orlikowski D., Bernholc J., Roland C., Nardelli M.B. (1999). Ad-dimers on Strained Carbon Nanotubes: A New Route for Quantum Dot Formation?. Phys. Rev. Lett..

[30] Fuentes M.L.C., Soto R.R. (2018). Carbon Nanotubes: Molecular and Electronic Properties of Regular and Defective Structures. Density Funct. Calc..

[31] Pakdel M., Raissi H., Shahabi M. (2019). Predicting doxorubicin drug delivery by single-walled carbon nanotube through cell membrane in the absence and presence of nicotine molecules: A molecular dynamics simulation study. J. Biomol. Struct. Dyn..

[32] Contreras M.L., Torres C., Villarroel I., Rozas R. (2019). Molecular dynamics assessment of doxorubicin–carbon nanotubes molecular interactions for the design of drug delivery systems. Struct. Chem..

[33] Torres C., Villarroel I., Rozas R., Contreras L. (2019). Carbon Nanotubes Having Haeckelite Defects as Potential Drug Carriers. Molecular Dynamics Simulation. Molecules.

[34] Zhang L., Peng G., Li J., Liang L., Kong Z., Wang H., Jia L., Wang X., Zhang W., Shen J.-W. (2018). Molecular dynamics study on the configuration and arrangement of doxorubicin in carbon nanotubes. J. Mol. Liq..

[35] Izadyar A.S., Farhadian N., Chenarani N. (2015). Molecular dynamics simulation of doxorubicin adsorption on a bundle of functionalized CNT. J. Biomol. Struct. Dyn..

[36] Sornmee P., Rungrotmongkol T., Saengsawang O., Arsawang U., Remsungnen T., Hannongbua S. (2011). Understanding the Molecular Properties of Doxorubicin Filling Inside and Wrapping Outside Single-Walled Carbon Nanotubes. J. Comput. Theor. Nanosci..

[37] Wolski P., Nieszporek K., Panczyk T. (2020). Carbon nanotubes and short cytosine-rich telomeric DNA oligomers as platforms for controlled release of doxorubicin-a molecular dynamics study. Int. J. Mol. Sci..

[38] Maleki R., Afrouzi H.H., Hosseini M., Toghraie D., Piranfar A., Rostami S. (2020). pH-sensitive loading/releasing of doxorubicin using single-walled carbon nanotube and multi-walled carbon nanotube: A molecular dynamics study. Comput. Methods Programs Biomed..

[39] Pennetta C., Floresta G., Graziano A.C.E., Cardile V., Rubino L., Galimberti M., Rescifina A., Barbera V. (2020). Functionalization of Single and Multi-Walled Carbon Nanotubes with Polypropylene Glycol Decorated Pyrrole for the Development of Doxorubicin Nano-Conveyors for Cancer Drug Delivery. Nanomaterials.

[40] Kordzadeh A., Amjad-Iranagh S., Zarif M., Modarress H. (2019). Adsorption and encapsulation of the drug doxorubicin on covalent functionalized carbon nanotubes: A scrutinized study by using molecular dynamics simulation and quantum mechanics calculation. J. Mol. Graph. Model..

[41] Karnati K.R., Wang Y. (2018). Understanding the co-loading and releasing of doxorubicin and paclitaxel using chitosan functionalized single-walled carbon nanotubes by molecular dynamics simulations. Phys. Chem. Chem. Phys..

[42] Wolski P., Nieszporek K., Panczyk T. (2017). Pegylated and folic acid functionalized carbon nanotubes as pH controlled carriers of doxorubicin. Molecular dynamics analysis of the stability and drug release mechanism. Phys. Chem. Chem. Phys..

[43] Rungnim C., Rungrotmongkol T., Poo-Arporn R.P. (2016). pH-controlled doxorubicin anticancer loading and release from carbon nanotube noncovalently modified by chitosan: MD simulations. J. Mol. Graph. Model..

[44] Janas D. (2020). From Bio to Nano: A Review of Sustainable Methods of Synthesis of Carbon Nanotubes. Sustain. J. Rec..

[45] Wang J., Cieplak P., Kollman P.A. (2000). How well does a restrained electrostatic potential (RESP) model perform in calculating conformational energies of organic and biological molecules?. J. Comput. Chem..

[46] Veclani D., Tolazzi M., Melchior A. (2020). Molecular Interpretation of Pharmaceuticals’ Adsorption on Carbon Nanomaterials: Theory Meets Experiments. Processes.

[47] Johnson E.R., Keinan S., Mori-Sánchez P., Contreras-García J., Cohen A.J., Yang W. (2010). Revealing Noncovalent Interactions. J. Am. Chem. Soc..

[48] Khalak Y., Tresadern G., de Groot B.L., Gapsys V. (2021). Non-equilibrium approach for binding free energies in cyclodextrins in SAMPL7: Force fields and software. J. Comput. Mol. Des..

[49] Westermaier Y., Ruiz-Carmona S., Theret I., Perron-Sierra F., Poissonnet G., Dacquet C., Boutin J.A., Ducrot P., Barril X. (2017). Binding mode prediction and MD/MMPBSA-based free energy ranking for agonists of REV-ERBα/NCoR. J. Comput. Mol. Des..

[50] Chéron N., Shakhnovich E.I. (2017). Effect of sampling on BACE-1 ligands binding free energy predictions via MM-PBSA calculations. J. Comput. Chem..

[51] Eken Y., Almeida N.M.S., Wang C., Wilson A.K. (2021). SAMPL7: Host–guest binding prediction by molecular dynamics and quantum mechanics. J. Comput. Mol. Des..

[52] Ruangpornvisuti V. (2009). Molecular modeling of dissociative and non-dissociative chemisorption of nitrosamine on close-ended and open-ended pristine and Stone-Wales defective (5,5) armchair single-walled carbon nanotubes. J. Mol. Model..

[53] Peng C., Wang J., Yu Y., Wang G., Chen Z., Xu Z., Cai T., Shao Q., Shi J., Zhu W. (2019). Improving the accuracy of predicting protein–ligand binding-free energy with semiempirical quantum chemistry charge. Futur. Med. Chem..

[54] Contreras M.L., Ávila D., Alvarez J., Rozas R. (2012). Computational algorithms for fast-building 3D carbon nanotube models with defects. J. Mol. Graph. Model..

[55] (2004). HyperChem Release.

[56] Case D.A., Betz R.M., Cerutti D.S., Cheatham T.E., Darden T.A., Duke R.E., Giese T.J., Gohlke H., Goetz A.W., Homeyer  N. (2016). AMBER 2016.

[57] Case D.A., Cheatham T.E., Darden T., Gohlke H., Luo R., Merz K.M., Onufrie A., Simmerling C., Wang B., Woods R. (2005). The Amber biomolecular simulation programs. J. Comput. Chem..

[58] Bayly C.I., Cieplak P., Cornell W., Kollman P.A. (1993). A well-behaved electrostatic potential based method using charge restraints for deriving atomic charges: The RESP model. J. Phys. Chem..

[59] Genheden S., Ryde U. (2015). The MM/PBSA and MM/GBSA methods to estimate ligand-binding affinities. Expert Opin. Drug Discov..

