# Supplementary material for: Doxorubicin Encapsulation in Carbon Nanotubes Having Haeckelite or Stone–Wales Defects as Drug Carriers: A Molecular Dynamics Approach
Source: Molecules. 2021 Mar 13;26(6):1586. doi: 10.3390/molecules26061586 (PMC7999666; doi:10.3390/molecules26061586)
Supplement: Supplementary file 1 [file molecules-26-01586-s001.pdf]

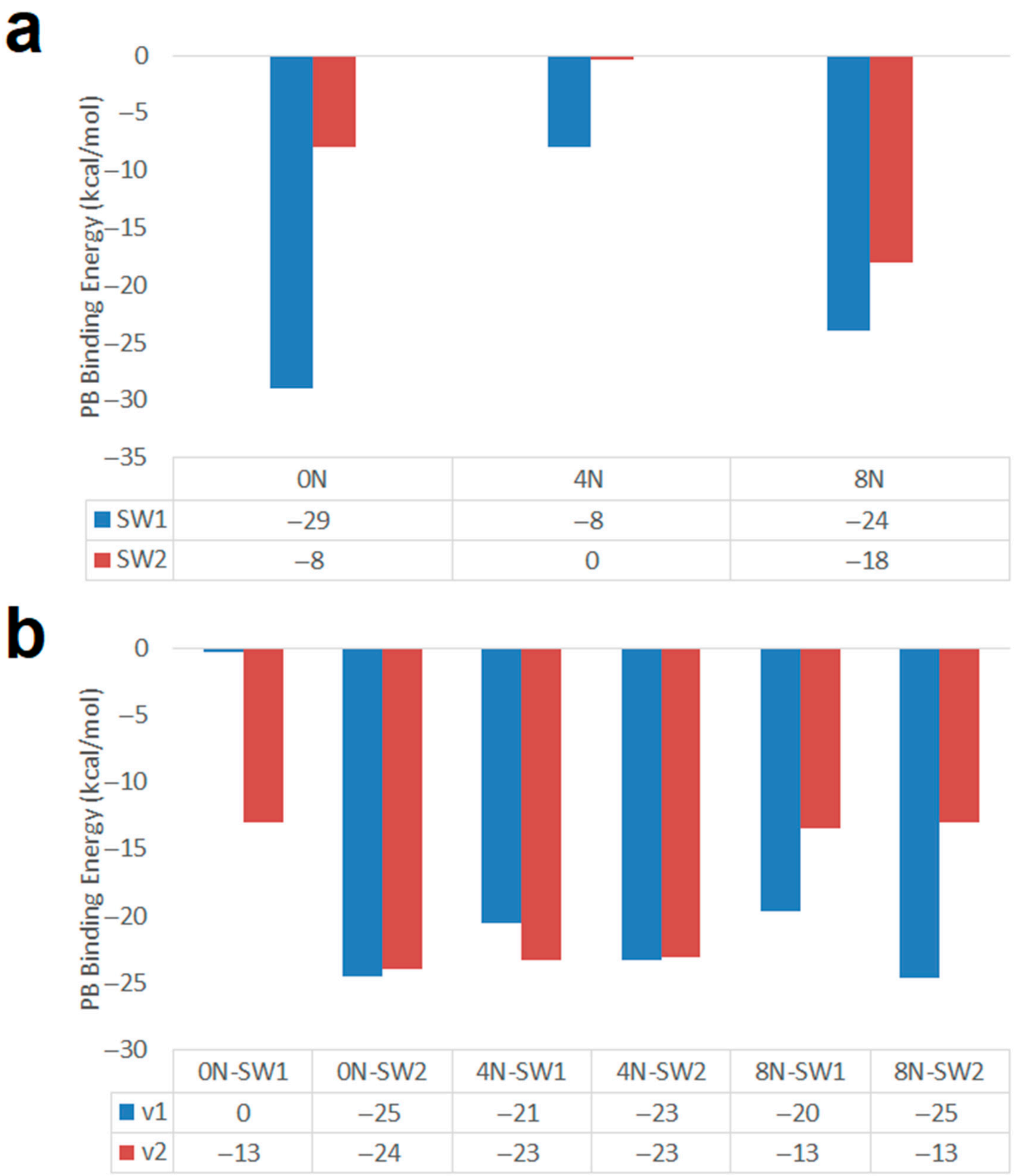


**Figure S1.** DOX-CNT PB relative binding energies for nitrogen-doped and undoped (10,10) *armchair* nanotubes of different length having one and two Stone-Wales defects. (a) 20 Å length; reference: 4N doped SW2 nanotube with –110 kcal/mol PB DOX-CNT binding energy; (b) 34 Å length; reference: 0N SW1 nanotube with –105 kcal/mol PB DOX-CNT binding energy.

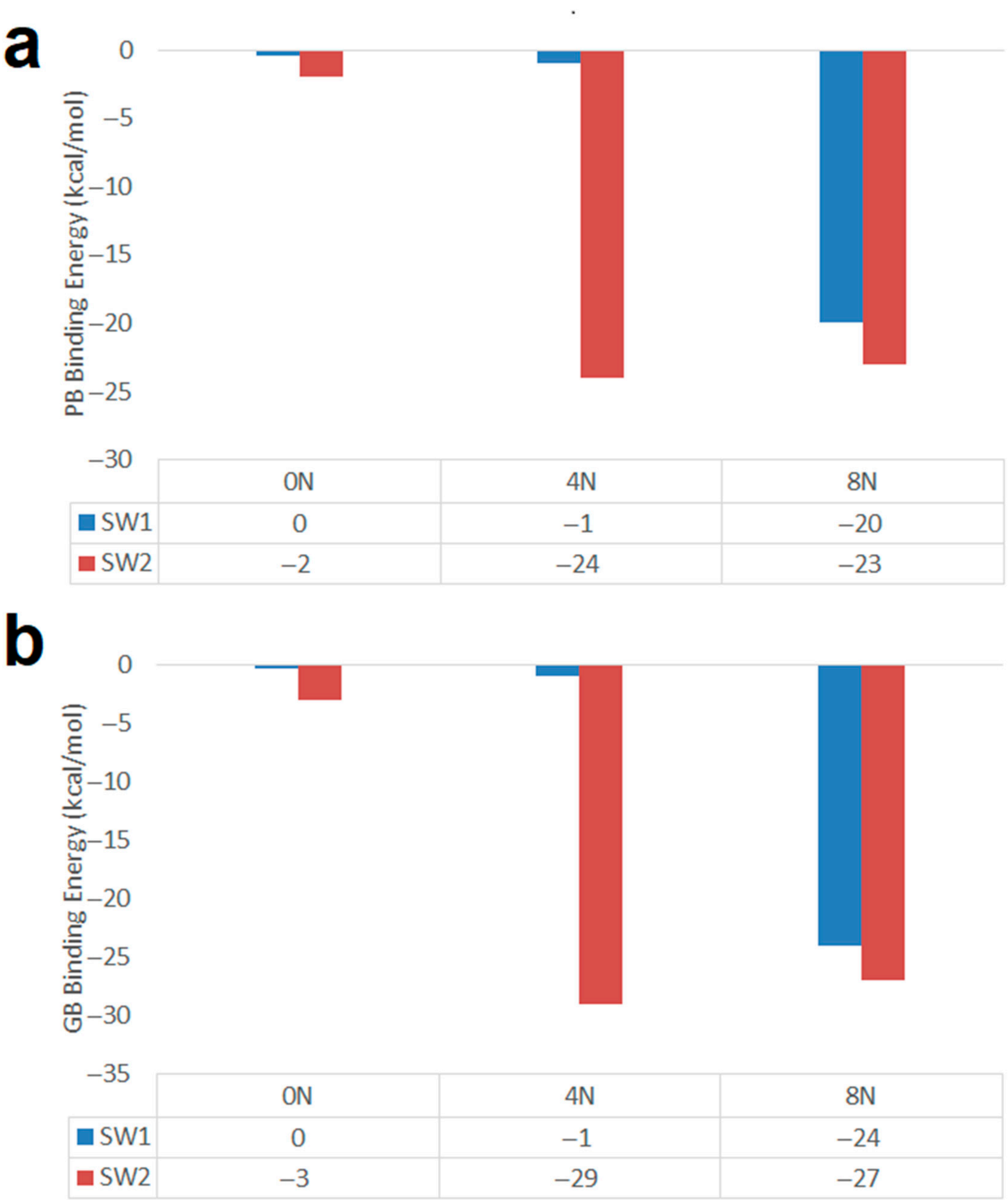


**Figure S2.** Representation of the PB and GB DOX-CNT relative binding energies for nitrogen-doped and undoped (18,0) *zigzag* nanotubes having one and two Stone-Wales defects. (a) PB binding energy. Reference: 0N SW1 nanotube with –102 kcal/mol PB DOX-CNT binding energy; (b) GB binding energy. Reference: 0N SW1 nanotube with –107 kcal/mol GB DOX-CNT binding energy.
